# Roundup and glyphosate’s impact on GABA to elicit extended proconvulsant behavior in *Caenorhabditis* *elegans*

**DOI:** 10.1038/s41598-022-17537-w

**Published:** 2022-08-23

**Authors:** Akshay S. Naraine, Rebecca Aker, Isis Sweeney, Meghan Kalvey, Alexis Adams, Venkatesh Shanbhag, Ken Dawson-Scully

**Affiliations:** 1 Department of Biological Sciences, Florida Atlantic University, Boca Raton, FL USA; 2 IMPRS for Synapses and Circuits, Max Planck Florida Institute for Neuroscience, Jupiter, FL USA; 3 Department of Chemistry and Physics, Halmos College of Arts and Sciences, Nova Southeastern University, Davie, FL USA; 4 Department of Psychology and Neuroscience, College of Psychology, Nova Southeastern University, Davie, FL USA

**Keywords:** Neuromuscular junction, Behavioural ecology, Drug safety

## Abstract

As 3 billion pounds of herbicides are sprayed over farmlands every year, it is essential to advance our understanding how pesticides may influence neurological health and physiology of both humans and other animals. Studies are often one-dimensional as the majority examine glyphosate by itself. Farmers and the public use commercial products, like Roundup, containing a myriad of chemicals in addition to glyphosate. Currently, there are no neurological targets proposed for glyphosate and little comparison to Roundup. To investigate this, we compared how glyphosate and Roundup affect convulsant behavior in *C.* *elegans* and found that glyphosate and Roundup increased seizure-like behavior. Key to our initial hypothesis, we found that treatment with an antiepileptic drug rescued the prolonged convulsions. We also discovered over a third of nematodes exposed to Roundup did not recover from their convulsions, but drug treatment resulted in full recovery. Notably, these effects were found at concentrations that are 1,000-fold dilutions of previous findings of neurotoxicity, using over 300-fold less herbicide than the lowest concentration recommended for consumer use. Exploring mechanisms behind our observations, we found significant evidence that glyphosate targets GABA-A receptors. Pharmacological experiments which paired subeffective dosages of glyphosate and a GABA-A antagonist yielded a 24% increase in non-recovery compared to the antagonist alone. GABA mutant strain experiments showed no effect in a GABA-A depleted strain, but a significant, increased effect in a glutamic acid decarboxylase depleted strain. Our findings characterize glyphosate’s exacerbation of convulsions and propose the GABA-A receptor as a neurological target for the observed physiological changes. It also highlights glyphosate’s potential to dysregulate inhibitory neurological circuits.

## Introduction

Glyphosate targets plant-specific amino acid synthesis pathways, specifically it reduces the conversion of shikimate to chorismite by inhibiting a key enzyme, 5-enolpyruvylshikimate-3-phosphate synthase^1,2^. Since this pathway is not shared between plants and animals, glyphosate has been proposed to be nontoxic to animals^2^. However, there is a wealth of data to suggest that the toxicity mechanisms are not that straightforward, and there is a high demand for independent research into the health effects of glyphosate-based herbicides^3^. One important distinction is that commercial herbicides are a mixture of chemicals; Roundup Super Concentrate is 50.2% glyphosate, and the other 49.8% is the adjuvant. The adjuvant improves the effectiveness of the active ingredient and is composed of surfactants, buffers, and emulsifiable oil activators^4^. One of these adjuvant surfactants, polyethoxylated tallowamine (POEA), has been found to confer toxicity in a host of invertebrate, aquatic, and mammalian models^5–10^. Exposure of human cells to ethoxylated adjuvants also resulted in significant toxicity^11^; both POEA and Roundup were found to affect intestinal muscle activity. Particularly, POEA exhibited high toxicity toward smooth muscle^12^. In 2016, the European Union placed a ban on POEA. While it was noted that POEA presented a number of risks, it was not found to have any neurotoxic effects^13^. In the United States, no similar legislative measures have been taken, thus it stands to reason that POEA, or a comparable surfactant is in the current US formulation of Roundup. Many studies have not directly compared the effects of Roundup to those of glyphosate; those that have found that Roundup induces higher levels of cytotoxicity and DNA damage compared to glyphosate in human cell lines^14–17^. Even fewer studies have investigated the neurotoxicity attributed to glyphosate and Roundup exposure. A potential reason why many studies focus on glyphosate is because the adjuvant components of Roundup are a trade secret. Since these co-formulants are not disclosed to the public, pinpointing chemicals, other than glyphosate, can be a significant challenge. Roundup is what is used agriculturally and domestically, so even though the co-formulants are rarely disclosed, investigating both glyphosate and Roundup is essential to factor in the potential effects of the adjuvant^10,18^. With glyphosate use projected to increase 200-fold in the US and similarly dramatic increases globally^19^, it is essential to understand the nuanced affects that may be influencing neurological effects.

*C.* *elegans*, a soil-dwelling nematode, presents a useful environmental and translational model for furthering our understanding of herbicides like Roundup. This translational importance was highlighted when one study found that the order of neurotoxicity induced by organophosphates in mammals was highly correlated to the order of toxicity in *C.* *elegans*^20^. As *C.* *elegans* has a conserved cellular physiology of much of the mammalian nervous system^21^, it has been used as a model to study glyphosate-based herbicides. Recent findings determined that exposure to a glyphosate-based herbicide led to degradation of dopaminergic and GABAergic neurons at 10% and 7% concentrations of glyphosate respectively^22,23^. Coordinated GABA neurotransmission is essential to the normal wave-like movement in *C.* *elegans,* and disruption to this coordination results in a seizure-like phenotype^24^. *C.* *elegans* GABA mutants have been characterized to refine mechanisms. For instance, *unc-*25(CB156) is a mutant deficient in glutamic acid decarboxylase(GAD)^25,26^, and *unc*-49(CB382) is deficient in the GABA-A receptor^25,27^. Due to GABA providing inhibitory input into the *C.* *elegans* motor system, these mutants have been tested in an electroconvulsive assay and were found to have extended convulsion duration compared to wildtype. In the same study, the inclusion of a GABA-A antagonist, pentylenetetrazol (PTZ), with the *unc-*25 mutant elicited extended convulsion duration from baseline^28^. In addition to this, it has been found that high temperatures induce seizure activity in *C.* *elegans*^29^, and with the effects of climate change an ever-present concern, it is essential to understand if herbicides affect soil-dwelling microorganisms. Because *C.* *elegans* has been used to study ictogenesis and seizure physiology^28,30,31^, we used an electroshock assay to probe the convulsant effects of Roundup and glyphosate with *C.* *elegans* as our screening model.

Due to the distinction between nuanced physiological, behavioral effects and toxicity, it was essential to examine dosages that were far lower than those which induce neurodegeneration and provide reasonable exposure to *C.* *elegans*. Since the Environmental Protection Agency advises label usage of Roundup, which is around 2% glyphosate for domestic use, we used a dosage of 0.1 mM, or about 0.002% glyphosate, to maintain physiological relevance and enhance potential translatability. We first tested glyphosate alone and both the US and UK formulations of Roundup from two distinct time periods, before and after the ban on POEA. These conditions were chosen to pinpoint which effects are specific to the active ingredient, Roundup formulations in general, the POEA surfactant, or any combination of these. In addition, we used an FDA approved antiepileptic drug (AED), sodium valproate (NaVal), which increases GABA neurotransmission. The AED treatment condition played an essential role in teasing out whether the GABAergic tracts were degraded and if any prolonged convulsions could be rescued by AED intervention. We then took a pharmacological approach and genetic approach to interrogate a potential mechanism. We used previously established subeffective dosages of PTZ^28^, a GABA-A antagonist, in conjunction with a subeffective dosage of glyphosate to detect possible synergistic effects. In support of the pharmacological experiments, *unc-*25 and *unc-*49 genetic mutant strains were used to investigate GAD and GABA-A receptors as potential targets.

## Results

### Proconvulsant effects of glyphosate mitigated by GABAergic antiepileptic drug

**Figure 1:**
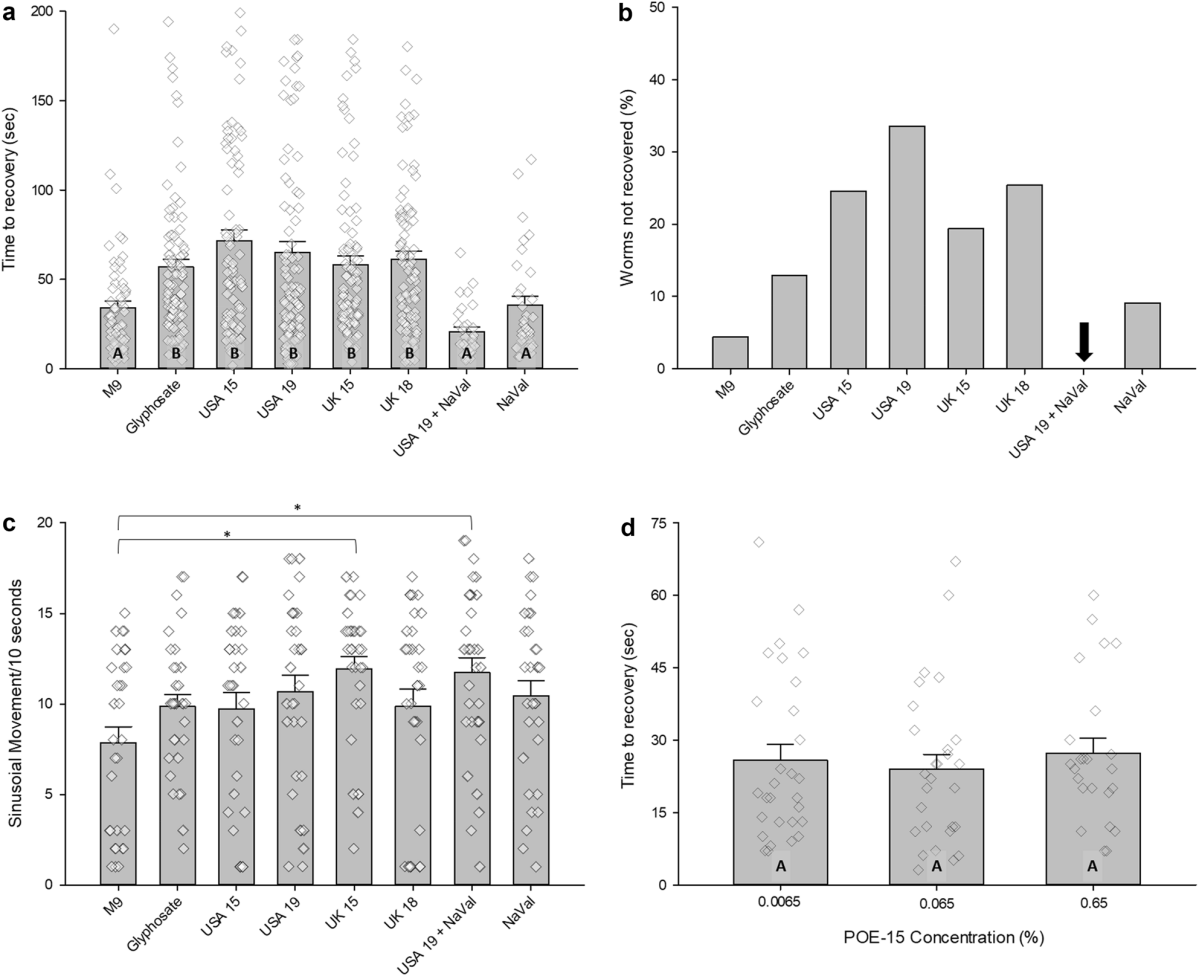
**(a**) Average time to recovery for N2-wildtype nematodes, approximately 100 worms per condition, exposed to M9 saline (n = 65), glyphosate (n = 85), US formulations of Roundup from 2015 (n = 85) and 2019 (n = 104), UK formulations of Roundup from 2015 (n = 95) and 2018 (n = 96), the US formulation of Roundup from 2019 co-administered with 3 mM sodium valproate (n = 31), and sodium valproate by itself (n = 33). Glyphosate-containing treatments were normalized to 0.1 mM glyphosate. Data presented as mean seconds to recovery ± SEM. Raw data points were overlayed on the histograms. Statistical significance (p < .05) was designated using letters, where different letters show statistically significant differences, and the same letter indicates non-significance. *P* values between indicated conditions derived from a One-Way ANOVA followed by Student–Newman–Keuls Method for all pairwise multiple comparisons. (**b**) Percent of worms that did not recover from convulsion state when exposed to M9 saline (n = 69), glyphosate (n = 116), US formulations of Roundup from 2015 (n = 114) and 2019 (n = 158), UK formulations of Roundup from 2015 (n = 119) and 2018 (n = 130), the US formulation of Roundup from 2019 co-administered with 3 mM sodium valproate (n = 31), and sodium valproate by itself (n = 36). Data presented as (number of worms not recovered in condition X/total number of worms tested in condition X) × 100. Arrow indicates that all worms recovered in the condition. (**c**) Average number of complete sinusoidal movements per 10 s before induction of electroshock. N2-wildtype nematodes were exposed to M9 saline (n = 31), glyphosate (n = 32), US formulations of Roundup from 2015 (n = 31) and 2019 (n = 33), UK formulations of Roundup from 2015 (n = 32) and 2018 (n = 33), the US formulation of Roundup from 2019 co-administered with 3 mM sodium valproate (n = 31), and sodium valproate by itself (n = 31). Data presented as mean sinusoidal movement/10 s time window ± SEM. Raw data points were overlayed on the histograms. Statistical significance (p < .05) was designated using stars. *P* values between indicated conditions derived from Kruskal–Wallis One-Way ANOVA on ranks followed by Dunn’s Method for multiple comparisons versus M9 control group. (**d**) Average time to recovery data for N2-wildtype exposed to logarithmic dosage of percent POE (15) tallow amine, 0.0065% (n = 29), 0.065% (n = 28), and 0.65% (n = 24). Data presented as mean seconds to recovery ± SEM. Raw data points were overlayed on the histograms. P = 0.588, *P* value derived from Kruskal–Wallis One-Way ANOVA on ranks.

Since GABA is critical for *C.* *elegans* locomotion, we used an electroshock convulsion assay at concentrations of 0.002% glyphosate to elucidate downstream physiological effects. *C.* *elegans* recovered in 34.1 s, on average, in normal saline conditions, but when exposed to glyphosate and Roundup formulations from the US and the UK, the convulsion duration spiked to 56.9 s or longer, an increase in seizure behavior of at least 66% (p < 0.01) (Fig. 1a). Exposure to the current Roundup formulation found in the US resulted in an average convulsion duration of 65.0 s, an increase of over 90% (p < 0.001). When the same formulation that generated a 90% increase in seizure behavior was co-administered with sodium valproate, an AED, durations of the spasms were rescued to control levels at 20.7 s, a drastic 68% decrease in convulsion duration (p < 0.001) (Fig. 1a).

While analyzing the convulsion duration data, we observed that several worms did not recover within the 5-min recording window. Less than 5.00% of worms did not recover in the M9 saline condition (Fig. 1b). Glyphosate exposure resulted in a jump to 12.9%, but in the Roundup conditions between 19.0 and 33.0% of worms did not recover from their seizure-like behavior within 5 min (Fig. 1b). The most extreme condition tested was the current Roundup formulation from the US at 33.5%, but when the AED was co-administered with Roundup, every worm recovered to normal locomotion (Fig. 1b). To test whether the herbicidal conditions were affecting locomotion before the electroshock was administered, the baseline swimming behavior was assessed. Apart from the 2015 Roundup formulation from the UK and the AED rescue condition, none of the conditions’ baseline locomotion differed from the M9 saline control (Fig. 1c). The UK 2015 and AED rescue conditions did not differ from the other herbicidal conditions (Fig. 1c).

POEA is a family of surfactant compounds^10^, and since the Roundup adjuvant component is a trade secret, we could only test one representative compound. POEA was banned in the UK in 2016 and the UK 2018 Roundup conditions still resulted in prolonged seizure behavior (Fig. 1a). Given this finding, we exposed *C.* *elegans* to a representative tallow amine surfactant, POE (15) tallow amine to further probe whether tallow amine surfactants affect seizure phenotype(methods). Of the three dosages of POE (15) tallow amine tested, none showed any increases in seizure behavior, and the times to recovery ranged between 23.9 and 27.2 s for all three conditions (Fig. 1d).

### Extended convulsion duration for glyphosate treated worms depends on dose

**Figure 2:**
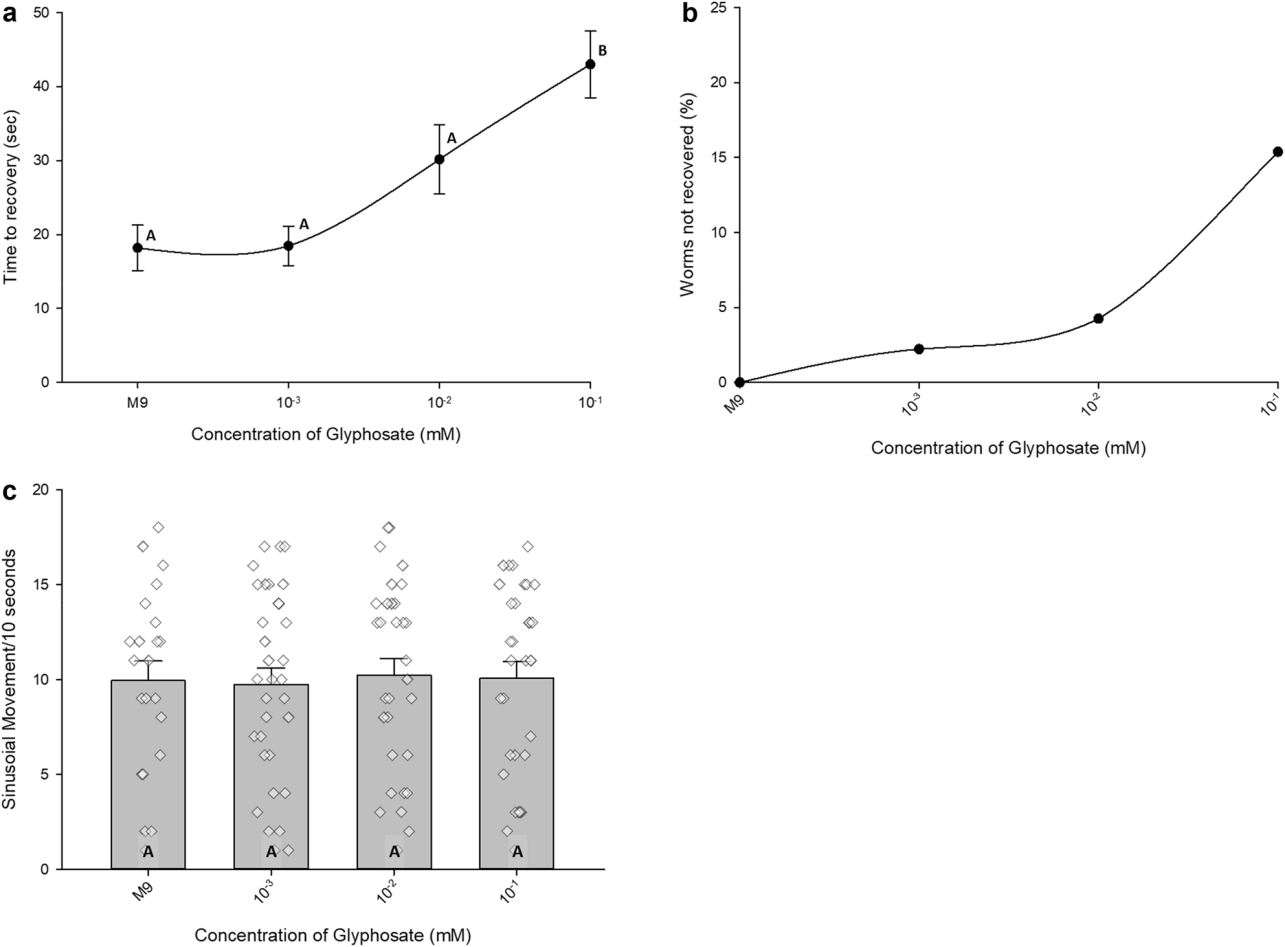
**(a)** Time to recovery for N2 nematodes exposed to logarithmic concentrations of glyphosate at 0 mM-M9 saline (n = 41), 0.001 mM (n = 45), 0.01 mM (n = 47), and 0.1 mM (n = 52). All glyphosate dilutions were done in M9 saline. Data presented as average seconds to recovery ± SEM. Statistical significance between conditions (p < .05) was designated using letters, where different letters show statistically significant differences, and the same letter indicates non-significance. *P* values between indicated conditions derived from Kruskal–Wallis One-Way ANOVA on ranks followed by Dunn’s Method for all pairwise multiple comparisons. (**b**) Percent of worms that did not recover from seizure when exposed to logarithmic concentrations of glyphosate at 0 mM-M9 saline (n = 41), 0.001 mM (n = 46), 0.01 mM (n = 49), and 0.1 mM (n = 60). Data presented as (number of worms not recovered in condition X/total number of worms tested in condition X) × 100. All worms recovered from seizure in M9 saline condition (0 mM glyphosate). (**c**) Number of complete sinusoidal movements per 10 s before induction of electroshock. N2 nematodes were exposed to logarithmic concentrations of glyphosate at 0 mM-M9 saline (n = 22), 0.001 mM (n = 33), 0.01 mM (n = 31), and 0.1 mM (n = 32). Data presented as mean sinusoidal movement/10 s time window ± SEM. Raw data points were overlayed on the histograms. P = 0.978, *P* value derived from Kruskal–Wallis One-Way ANOVA on ranks.

Glyphosate elicited a similar increase in spasm duration to that of Roundup (Fig. 1a), therefore we implemented logarithmic dilutions of the starting concentration, 0.1 mM, to determine if the prolonged seizure behavior could be observed. At 10% of the starting concentration, the convulsion duration significantly dropped to 30.2 s (Fig. 2a). The further dilution and saline conditions did not present any significant decreases in seizure duration (Fig. 2a).

We also made similar observations that some worms did not recover from their spasms during the 5-min recording period. The dosage curve for recovery (Fig. 2b) revealed similar trends to the convulsion duration curve (Fig. 2a). In the saline and dilution conditions, no more than 4.30% of worms did not recover, but at the 0.1 mM condition, 15.4% of worms did not recover from their spasms, an increase in non-recovery of 250% (Fig. 2b). This echoes the originally reported instance of non-recovery (Fig. 1b). Additionally, we ensured that the glyphosate dosages were not affecting the baseline swimming behavior. We found no significant differences in the pre-shock swimming behavior in each of the conditions (Fig. 2c).

### GABA-A disruption essential to glyphosate-induced extended seizure behavior

**Figure 3:**
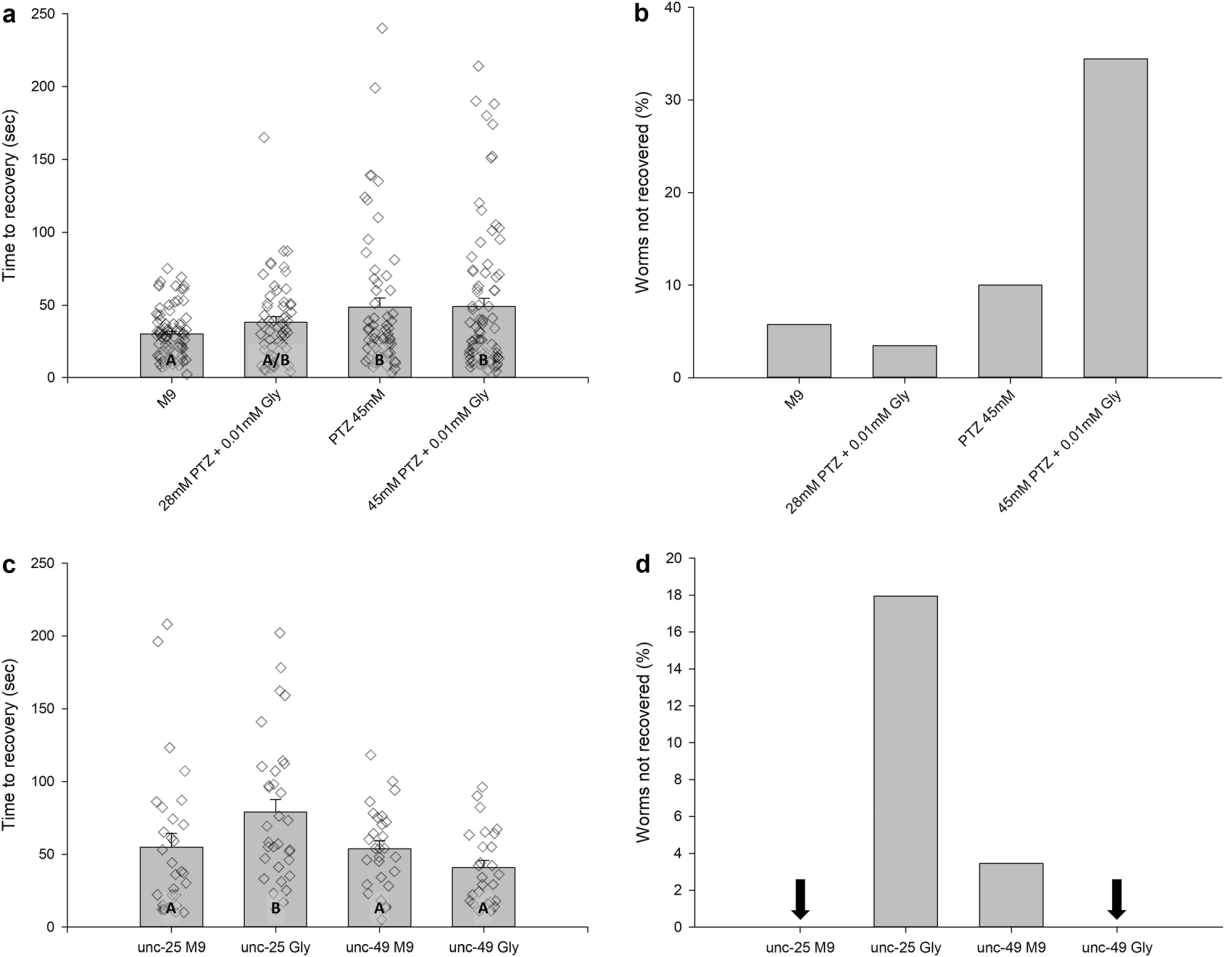
**(a**) Average time to recovery for N2-wildtype nematodes exposed to M9 saline (n = 82), 28 mM PTZ + 0.01 mM glyphosate (n = 56), 45 mM PTZ (n = 81), and 45 mM PTZ + 0.01 mM glyphosate (n = 59). Data presented as mean seconds to recovery ± SEM. Raw data points were overlayed on the histograms. Statistical significance (p < .05) was designated using letters, where different letters show statistically significant differences, and the same letter indicates non-significance. *P* values between indicated conditions derived from a One-Way ANOVA followed by Student–Newman–Keuls Method for all pairwise multiple comparisons. (**b**) Percent of worms that did not recover from convulsion state when exposed to M9 saline (n = 87), 28 mM PTZ + 0.01 mM glyphosate (n = 58), 45 mM PTZ (n = 90), and 45 mM PTZ + 0.01 mM glyphosate (n = 90). Data presented as (number of worms not recovered in condition X/total number of worms tested in condition X) × 100. (**c**) Average time to recovery for *unc*-25 nematodes exposed to M9 saline (n = 30) and 0.1 mM glyphosate (n = 32), and *unc*-49 mutant strain nematodes exposed to M9 saline (n = 28) and 0.1 mM glyphosate (n = 26). Data presented as mean seconds to recovery ± SEM. Raw data points were overlayed on the histograms. Statistical significance (p < .05) was designated using letters, where different letters show statistically significant differences, and the same letter indicates non-significance. *P* values between indicated conditions derived from a One-Way ANOVA followed by Student–Newman–Keuls Method for all pairwise multiple comparisons. (**d**) Percent of worms that did not recover from convulsion state for *unc*-25 nematodes exposed to M9 saline (n = 30) and 0.1 mM glyphosate (n = 39), and *unc*-49 mutant strain nematodes exposed to M9 saline (n = 29) and 0.1 mM glyphosate (n = 26). Data presented as (number of worms not recovered in condition X/total number of worms tested in condition X) × 100. Arrow indicates that all worms recovered in the condition.

Using subeffective dosages of 0.01 mM glyphosate (Fig. 2a,b) and of 45 mM and 28 mM PTZ^28^, we found a significant increase from M9 and 28 mM PTZ + 0.01 mM glyphosate to both conditions using 45 mM PTZ (p < 0.05) (Fig. 3a). The time to recovery data did not divulge any evidence of synergistic effects between PTZ and glyphosate, but when the recovery data were quantified, it revealed an 8.3% non-recovery in 45 mM PTZ compared to 34% non-recovery in 45 mM PTZ + 0.01 mM glyphosate (Fig. 3b).

The time to recovery baselines for *unc*-25, deficient in GAD, and *unc*-49, deficient in GABA-A receptors, in saline were established to be 54.9 and 53.8 s respectively (Fig. 3c). 0.1 mM glyphosate, the effective threshold for increased time to recovery and non-recovery phenotypes, increased *unc*-25 time to recovery to 78.9 s (p < 0.05) (Fig. 3c) and resulted in a non-recovery percentage of 17.9% (Fig. 3d). The effective dosage for glyphosate did not significantly impact *unc*-49. When exposed to glyphosate, the time to recovery decreased but remained comparable to the *unc*-49 baseline (ns) (Fig. 3c). All the *unc*-49 mutants exposed to 0.1 mM glyphosate recovered from the electroshock as well (Fig. 3d).

## Discussion

We have shown that exposure to glyphosate and Roundup results in proconvulsant behaviors and when treated with the FDA approved AED, sodium valproate, we remedied the extended convulsion durations. More than this, we found that herbicide exposure resulted in a concerning non-recovery phenotype, but the AED treatment rescued our most extreme non-recovery condition, exposure to a recent Roundup formulation from the US (p < 0.001). Based on our representative surfactant data, POE (15) tallow amine exposure did not affect seizure behavior. The 0.1 mM concentration of glyphosate seems to be a critical dosage for the effects observed in our electroshock assay, both for convulsion duration and non-recovery observations. To build upon previously reported findings of neurotoxicity and stress in *C.* *elegans*^22,23,32,33^, we provided some critical insights into prolonged locomotive abnormalities that result from exposure to glyphosate and Roundup. To our knowledge, Roundup and glyphosate have never been tested at our experimental concentration of 0.1 mM. While 7 and 10% concentrations are analogous to high concentrations that humans may acutely encounter, we have shown proconvulsant effects at a concentration of 0.002% which not only presents physiological relevance to our invertebrate model but also enhances the translational potential for understanding affects that may occur in humans. Building upon the sodium valproate findings, we probed GABAergic mechanisms using PTZ, a GABA-A antagonist, and found that the inclusion of a subeffective dosage of glyphosate tripled the non-recovery percentage. This provided substantial evidence of synergy between PTZ and glyphosate indicating the glyphosate may function similarly as a GABA-A antagonist. Using *unc*-25 mutant *C.* *elegans*, we did not find evidence that glyphosate targeted GAD to impact time to recovery or non-recovery as both phenotypes were substantially impacted. The *unc*-49 mutant findings demonstrated that by severely depleting GABA-A receptors, glyphosate’s impact on time to recovery and non-recovery were nullified.

We used *C.* *elegans* in this study to inform two distinct avenues: to contextualize the findings of herbicide exposure negatively affecting human health, and to determine the direct concerns to essential soil microorganisms. Herbicide exposure is linked to depression in humans, while insecticide and fungicide exposure are not^34^. Expanding past behavioral findings, herbicide use has been linked to increased risk of developing Parkinson’s Disease^35–40^. These findings lend themselves to two critical distinctions: there is evidence to further investigate how chronic exposure and accumulation may lead to neurodegenerative disease such as Parkinson’s Disease, but, critical to this study, there is also a sub-neurodegenerative threshold that may dramatically impact dysregulation of neurotransmission. In *C.* *elegans*, there is a concentration where exposure to a glyphosate-based herbicide is neurotoxic^22^, but GABAergic dysregulation, specifically deficits in GABAergic signaling, is a common pathophysiology in humans and rodent models with mood disorders such as anxiety disorder and depression^41–44^. Acute exposure at 0.002% glyphosate results in a significant behavioral and physiological effect. The evidence of GABA-A antagonism proposed as a result of this study prompts further investigation into both locomotive and behavioral parameters. Glyphosate has been found to cross the blood–brain barrier and increase its permeability^45^. Brain regions rich in GABA-A receptors, like the basal ganglia^46^, may be targeted as a result of glyphosate exposure. The nematode data indicates that there is an important distinction between exposure to glyphosate and Roundup resulting in behavioral effects versus outright toxicity or neurodegeneration. These behavioral effects are also detectable at levels of exposure thousands of times lower than those that induce toxicity. GABA-A receptors are also essential for locomotion. There was a published case study that showed that in infantile triplets, bioaccumulation of glyphosate seemed to play a role in the development of autism-like symptoms and seizure^47^. As of now, there is no information for how exposure to glyphosate and Roundup may affect humans diagnosed with epilepsy or other seizure disorders. The current study indicates that there is significant disruption in locomotion and should prompt further vertebrate studies.

Taken in an ecological light, Roundup has been found to affect the locomotion and nervous systems of macroscopic invertebrates such as earthworms and cockroaches^48,49^, and even increase soil nutrient concentrations^48^. Our non-recovery phenotype and prolonged convulsions in *C.* *elegans* set a foundation for understanding nuanced physiological effects that occur exponentially below neurotoxic levels. *C.* *elegans* undergo convulsions under thermal stress^29^, and our data strongly implicates glyphosate and Roundup exposure in exacerbating convulsive effects.

A challenge which remains is fully exploring chemical mechanisms behind our observations. The only known chemical in Roundup is glyphosate. POEA is a family of ethoxylated tallowamines, so even though we have used POE-15, we are not certain that POE-15 is included in the Roundup formulation. Understanding which chemical components are driving our observations is an essential undertaking. Many herbicidal formulations are trade secrets, and the unknown chemical components create enormous confounds for mechanistic studies^10,18^. It does not appear that this lack of chemical information impacted our findings as there was no significant difference between the convulsion duration of glyphosate and Roundup-treated animals. Additionally, the POEA representative compound did not affect seizure behavior, even at a concentration ten-times that which is found in a 0.1 mM herbicide concentration. Lastly, glyphosate, the active ingredient without co-formulants, drastically increased convulsion duration and nonrecovery in the *unc*-25 mutant, but time to recovery and nonrecovery remained unaffected for *unc*-49 mutants exposed to glyphosate. Thus indicating that glyphosate is the primary driver of both increased convulsion duration and nonrecovery findings.

To build upon these initial findings, we can translate our electroshock assay from *C.* *elegans* to the Maximal Electroshock Seizure Test^50–52^ in mammals to provide further insight into glyphosate’s influence on seizure-like behavior and physiology to begin to understand the potential neurological dangers to humans. It should be noted that the concern for human health is just one facet of the broader implications of our findings. *C.* *elegans* are soil-dwelling nematodes, so there is also a significant neuroethological perspective presented. The full exploration of our work is addressing both concerns to human health and the larger ecological perspective.

We show that glyphosate and Roundup, at a dosage over 300-fold less than that recommended for human exposure by the EPA, significantly extends seizure duration in an invertebrate model system and is remedied by the addition of an FDA approved AED. We also present significant evidence that glyphosate serves as a GABA-A antagonist. These physiological findings raise concerns over how pesticide exposure may affect those with epilepsy and seizure disorder, and the unique electroshock assay used in this study can be translated to higher-order rodent models to explore effects in mammalian vertebrates. Finding neurological effects at very dilute concentrations may also support the concern that herbicide exposure affects GABA-mediated mental health symptoms, particularly depression, in humans^34,43^. While high concentrations of 7% and 10% glyphosate may be necessary to induce acute toxicity^22,23^, an acute exposure at a concentration of 0.002% confers equally concerning and physiologically relevant behavioral effects. Our findings provide novel insight into the GABA-A receptor mediated proconvulsant effects of glyphosate and Roundup and generate concern over how herbicide use might affect soil-dwelling organisms like *C.* *elegans*.

## Methods

### *C. elegans* husbandry

*C*. *elegans* were maintained on standard NGM agar plates seeded with OP50 *E*. *coli*. L4 worms were picked and transferred to a fresh NGM plate with OP50 *E.* *coli* the evening prior to testing and maintained overnight at 25 °C. *C*. *elegans* used in these experiments were wild-type, Bristol N2 strain, *unc*-25(e156) III(CB156), and *unc*-49(e382) III(CB382). *C*. *elegans* strains were ordered from the Caenorhabditis Genetics Center (NIH Office of Research Infrastructure Programs, P40 OD010440).

### Electroshock convulsion assay

**Figure 4:**
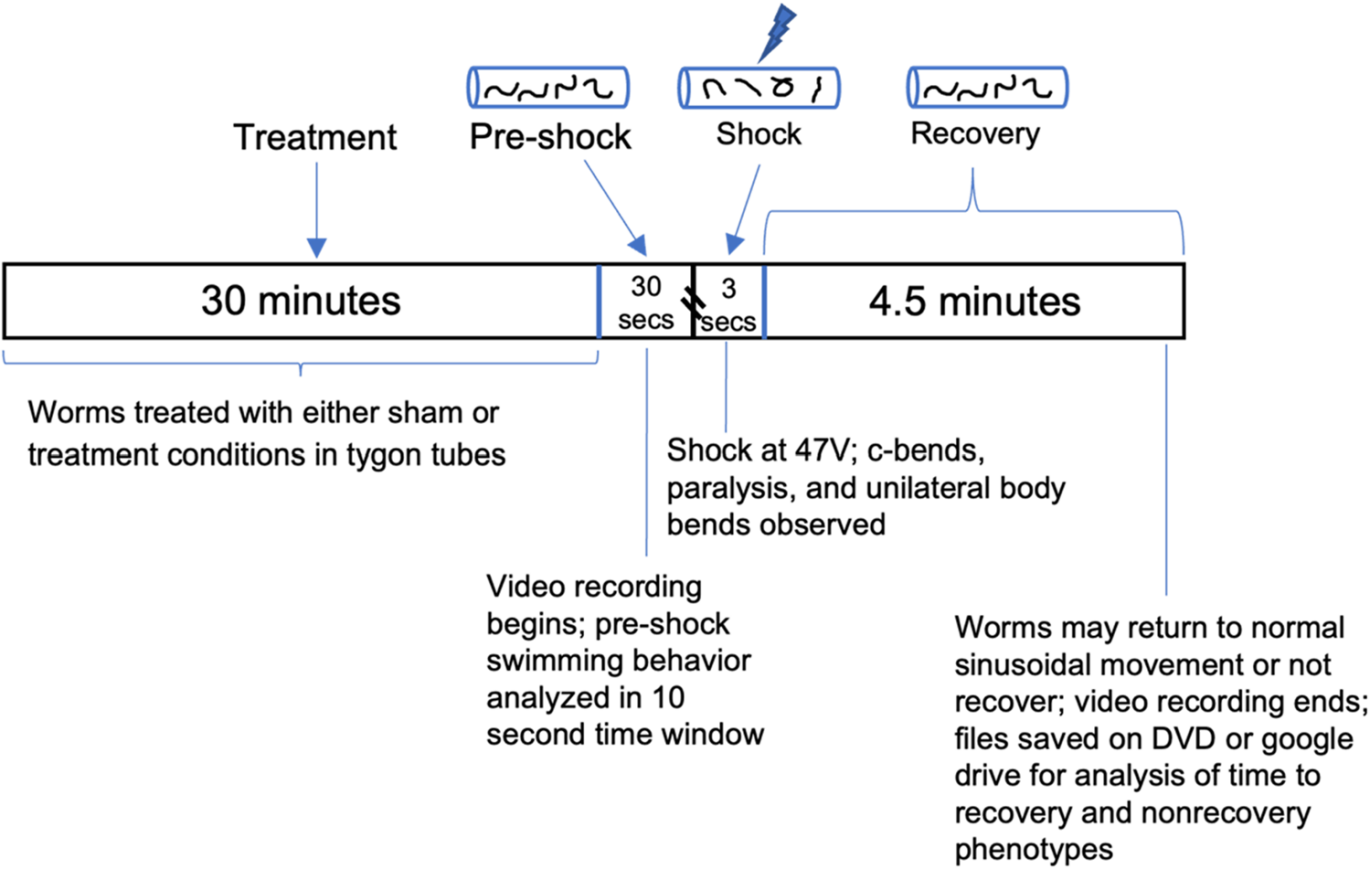
A detailed schematic of the timeline of electroshock assay data collection. Above the timeline are the four critical time parts of the assay. Below the timeline are details of each part and the crucial phenotypes that are observed.

On the day prior to experimentation, L4 stage *C*. *elegans* were selected and placed on an NGM agar plate with *OP50* *E*. *coli* and stored at 25 °C. Tygon^®^ microbore tubing (Taylor Scientific product number 13–9124-20) was cut into 9 mm segments and filled with 15μL of M9 solution or M9 + drug treatment. Thirty minutes prior to stimulation, about six, individual 1-day old adult *C*. *elegans* were picked directly off the NGM plate that was incubated overnight with L4s and transferred to the tube (Supp Fig. 3). For all treatments, worms were incubated for a total of 30 min. Following incubation, two copper wires were inserted approximately 2 mm into either end of the plastic tube. Two alligator clips were attached to the two copper wires and connected to a square-pulse generating stimulator (Grass SD9). The copper wires are measured to be about 1 cm apart inside the tube to maintain a constant electric field. A shock was delivered for 3 s (200 Hz, 3.5 ms, 47 V) and a dissecting microscope (10× magnification) with camera (Hitachi model KP-D20BU/4K Ultra HD HDMI C-mount Digital Microscope Camera) recorded at least 30 s before the shock and subsequent 5 min (Fig. 4). Experimental tubes were discarded after stimulation^28,30,53^.

### Convulsion analysis

After the data were recorded as videos on DVDs or a google drive, the experiments were visually analyzed, and times determined by video time marks. The time from the beginning of the shock to the time when each individual animal resumed a sinusoidal wave-like swimming motion (normalized as 3 consecutive sinusoidal undulations) was recorded. The speed of the sinusoidal wave was not taken into consideration when considering recovery, only the wave motion itself. As a result of electrolysis, peripheral bubbles formed on either end of the stimulation tube; *C*. *elegans* occluded by the peripheral bubbles were excluded from analysis. Animals who were initially moving and observed not to recover from the electroshock convulsion were not considered in recovery analysis, but they were analyzed separately. We also noted that a small number of animals did not move in the tubing at any point; these individuals were not considered in any of the analyses^28,30^. Each video and its relevant parameters (i.e., time recovery, sinusoidal movement, and nonrecovery) were analyzed by at least one researcher. 3 different researchers were involved in data analysis, and five different researchers collected data. No single figure was collected and analyzed by one researcher.

The novel non-recovery observation is included for *C.* *elegans* which initially had sinusoidal swimming behavior and did not recover any normal locomotion within the 5 min of video recording. These animals were not included in the time to recovery analysis in any way.

A subset of experimental trials was used to analyze pre-shock swimming behavior. The same 10-s window in all videos were taken between the 3 and 13 s time points for analysis, and each complete undulation, or sinusoidal movement, was visually counted.

### Antiepileptic drug and herbicidal treatment

For all the experimental conditions, glyphosate and Roundup were diluted in M9 saline. Glyphosate (Chem Service 8093800), Roundup Super Concentrate USA 2015 (derived from V.S.), Roundup Super Concentrate USA 2019 (Monsanto Company I18054), Roundup Super Concentrate UK 2015 (Monsanto UK Limited C5400), and Roundup Super Concentrate USA 2018 (Monsanto UK Limited C8102) were normalized to a concentration of 0.1 mM glyphosate (Fig. 1). Sodium Valproate (Sigma Aldrich S0930000) was dissolved in M9 saline to a concentration of 3mM^28^. POE (15) tallow amine has been used as a representative surfactant compound^7,10^ and was diluted to a level of approximate equivalence to the Roundup concentration dilution used (ChemService CAS: 61791-26-2). Glyphosate and POEA are the only two known chemicals in Roundup, but using the POE-15, glyphosate, and UK formulations, appropriate chemical controls are tested to achieve a robust screening of seizure behavior. Pentylenetetrazole (Sigma Aldrich CAS: 54-95-5) was dissolved in M9 Saline to concentrations of 28 mM and 45 mM. See Table 1 for a complied list of all conditions. The *C.* *elegans* were acutely incubated in the various conditions for 30 min prior to electroshock.

**Table 1:** All strains, conditions, and number of worms tested are organized by figure number and letter which corresponds to those in the main text.

Conditions Fig. 1a	Conditions Fig. 1b	Conditions Fig. 1c	Conditions Fig. 1d	Conditions Fig. 2a	Conditions Fig. 2b	Conditions Fig. 2c	Conditions Fig. 3a	Conditions Fig. 3b	Conditions Fig. 3c	Conditions Fig. 3d
Strain(s) used: N2	Strain(s) used: N2	Strain(s) used: N2	Strain(s) used: N2	Strain(s) used: N2	Strain(s) used: N2	Strain(s) used: N2	Strain(s) used: N2	Strain(s) used: N2	Strain(s) used: unc-25(e156) III (CB156)*,* unc-49(e382) III (CB382), N2 (Supp Fig. 1)	Strain(s) used: unc-25(e156) III (CB156)*,* unc-49(e382) III (CB382), N2 (Supp Fig. 2)
M9 saline (n = 65)	M9 saline (n = 69)	M9 saline (n = 31)	0.0065% POE-15 (n = 29)	M9 saline (n = 41)	M9 saline (n = 41)	M9 saline (n = 82)	M9 saline (n = 82)	M9 saline (n = 87)	unc-25 M9 (n = 30)	unc-25 M9 (n = 30)
Glyphosate (n = 85)	Glyphosate (n = 116)	Glyphosate (n = 32)	0.065% POE-15 (n = 28)	0.001mM glyphosate (n = 45)	0.001mM glyphosate (n = 46)	0.001mM glyphosate (n = 33)	28mM PTZ + 0.01mM glyphosate (n = 56)	28mM PTZ + 0.01mM glyphosate (n = 58)	unc-25 in 0.1mM glyphosate (n = 32)	unc-25 in 0.1mM glyphosate (n = 39)
US formula 2015 (n = 85)	US formula 2015 (n = 114)	US formula 2015 (n = 31)	0.65% POE-15 (n = 24)	0.01mM glyphosate (n = 47)	0.01mM glyphosate (n = 49)	0.01mM glyphosate (n = 31)	45mM PTZ (n = 81)	45mM PTZ (n = 90)	unc-49 M9 (n = 28)	unc-49 M9 (n = 29)
US formula 2019 (n = 104)	US formula 2019 (n = 158)	US formula 2019 (n = 33)		0.1mM glyphosate (n = 52)	0.1mM glyphosate (n = 60)	0.1mM glyphosate (n = 32)	45mM PTZ + 0.01mM glyphosate (n = 59)	45mM PTZ + 0.01mM glyphosate (n = 90)	unc-49 in 0.1mM glyphosate (n = 26)	unc-49 in 0.1mM glyphosate (n = 26)
UK formula 2015 (n = 95)	UK formula 2015 (n = 119)	UK formula 2015 (n = 32)								
UK formula 2018 (n = 96)	UK formula 2018 (n = 130)	UK formula 2018 (n = 33)								
US 2019 formula + 3mM sodium valproate (n = 31)	US 2019 formula + 3mM sodium valproate (n = 31)	US 2019 formula + 3mM sodium valproate (n = 31)								
3mM sodium valproate (n = 33)	3mM sodium valproate (n = 36)	3mM sodium valproate (n = 31)								

All Roundup conditions standardized to 0.1mM glyphosate for testing.

Some of glyphosate and Roundup’s toxicity has been attributed to pH effects, as more concentrated doses are acidic^20,54^. We checked the pH of our Roundup dilutions to ensure that any proconvulsant behaviors were attributed to pH effects. We found the M9 saline to have an average pH of 6.37, while the Roundup US 2019 had a comparable pH of 6.51. The AED + Roundup rescue condition was found to have a pH of 6.44.

### Statistical analysis

No statistical methods were used to predetermine sample size. Statistical analysis was conducted using SigmaPlot (v11). For the multiple comparisons testing, one-way ANOVAs were used followed by Dunn’s Method and Student–Newman–Keuls Method post hoc test. All pairwise comparisons were conducted for significant ANOVAs, except for the pre-shock swimming behavior which analyzed conditions versus control (Fig. 1c). Data are represented as mean ± SEM, and the significance was set at *P* < 0.05.

## Supplementary Information

Supplementary Figure 1.

Supplementary Figure 2.

Supplementary Figure 3.

## Data Availability

The datasets generated and/or analyzed during the current study are available from the corresponding author upon reasonable request.
