# Supplementary figures and images for: Roundup and glyphosate’s impact on GABA to elicit extended proconvulsant behavior in Caenorhabditis elegans
Source: Sci Rep. 2022 Aug 23;12:13655. doi: 10.1038/s41598-022-17537-w (PMC9399239; doi:10.1038/s41598-022-17537-w)

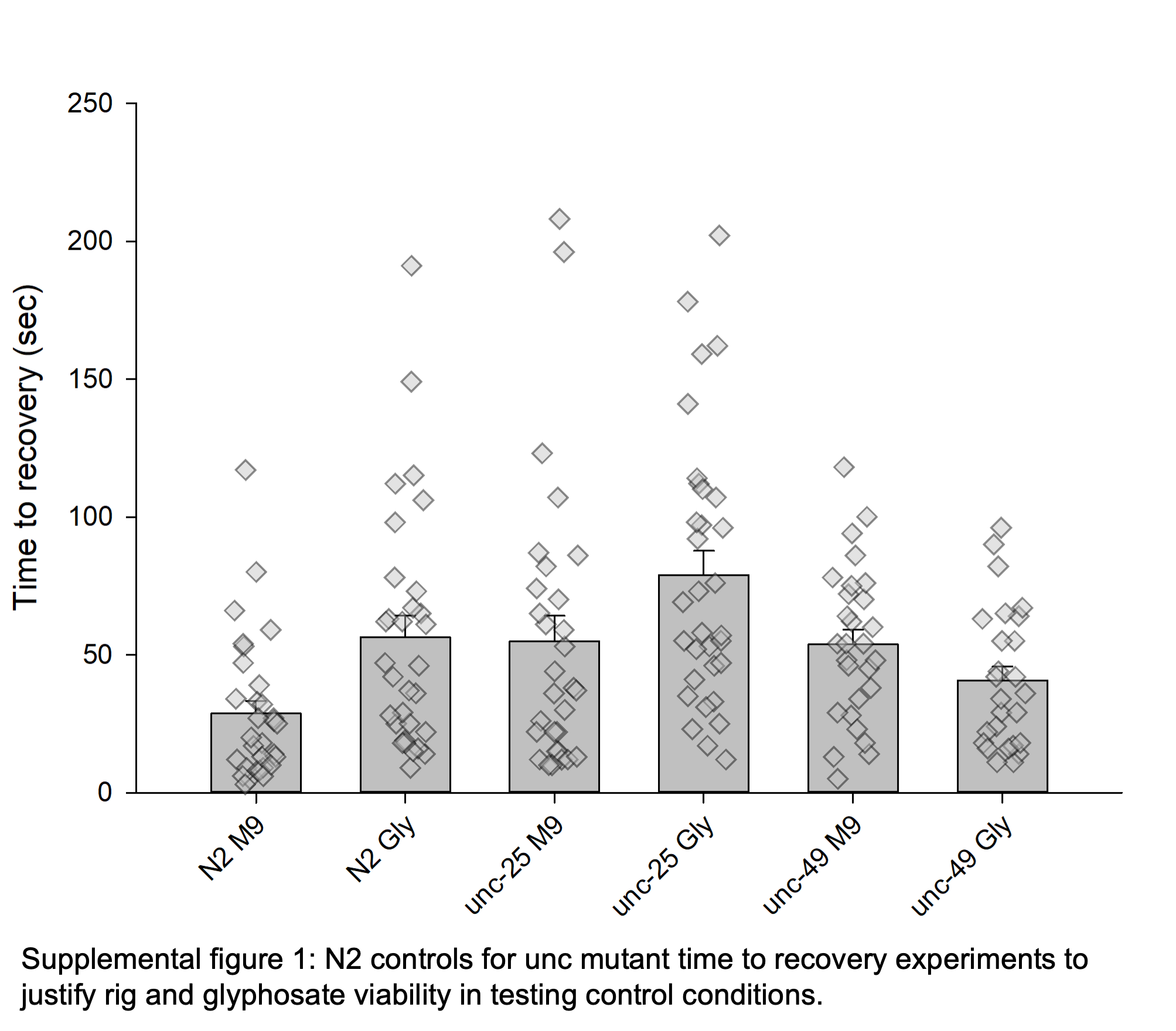

Supplement: Supplementary file 1 — Supplementary Figure 1. [file 41598_2022_17537_MOESM1_ESM.png]

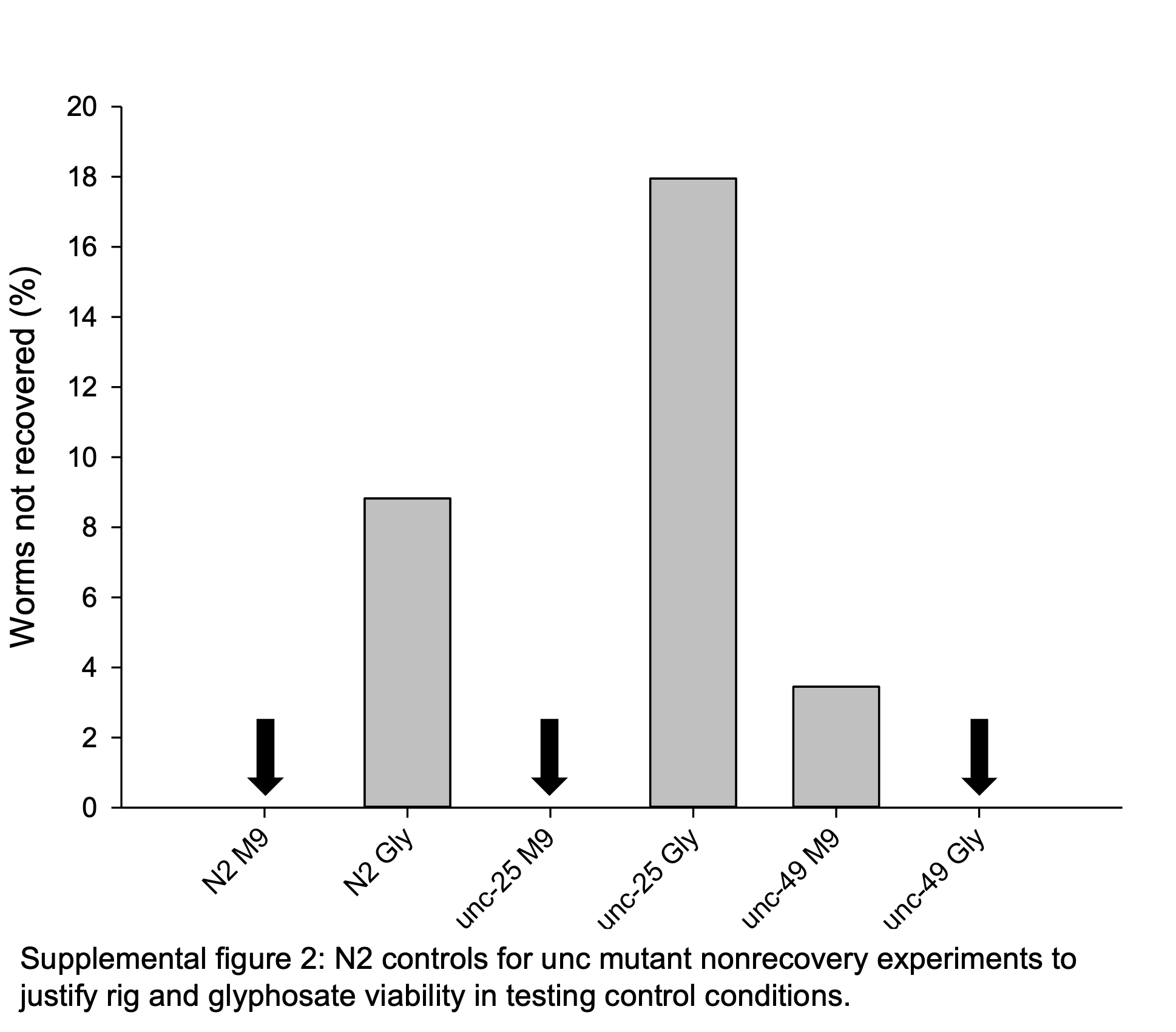

Supplement: Supplementary file 2 — Supplementary Figure 2. [file 41598_2022_17537_MOESM2_ESM.png]

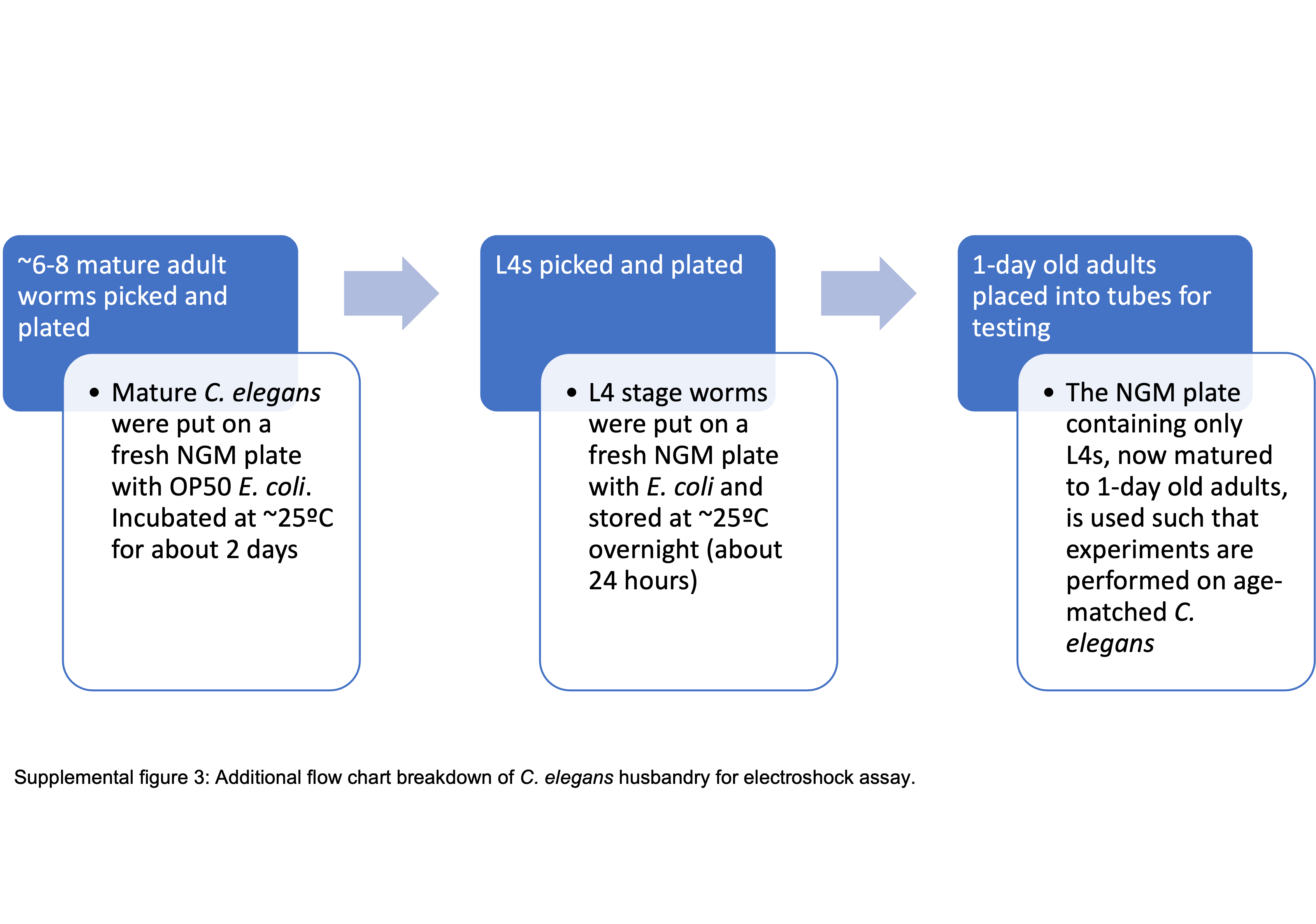

Supplement: Supplementary file 3 — Supplementary Figure 3. [file 41598_2022_17537_MOESM3_ESM.png]
